# Addressing Complications in Cardiac Implantable Electronic Devices: A Guideline to Prevention of CIED Infection

**DOI:** 10.3390/jcdd12100406

**Published:** 2025-10-13

**Authors:** Benito Baldauf, Roberto Cemin, Mauro Biffi, Antonio Rapacciuolo, Giulio Zucchelli, Maria Grazia Bongiorni, Ernesto Casorelli, Gianfranco Mitacchione, Felix Hohendanner, Emanuele Durante-Mangoni, Veronica Dusi, Paul William Xavier Foley, Angelo Pan, Giuseppe Arena, Archana Rao, Sebastian Spencker, Alexander Steger, Carlo Tascini, Valerio Zacà, Federico Migliore, Ojan Assadian, Marzia Giaccardi, Hendrik Bonnemeier, Kerstin Bode

**Affiliations:** 1 Medical Faculty, Christian-Albrechts University, Christian-Albrechts-Platz 4, 24118 Kiel, Germany; bonnemeier@t-online.de; 2 Institute of Life Sciences, University of Applied Sciences, An der Karlstadt 8, 27568 Bremerhaven, Germany; 3 Department of Cardiology, ICU, Ospedale Regionale San Maurizio, Via Lorenz Böhler 5, 39100 Bolzano, Italy; roberto.cemin@sabes.it; 4 Institute of Cardiology, IRCCS Azienda Ospedaliero-Universitaria di Bologna, Via Massarenti 9, 40138 Bologna, Italy; mauro.biffi@aosp.bo.it; 5 Department of Advanced Biomedical Sciences, University of Naples Federico II, Via Sergio Pansini, 80131 Naples, Italy; antonio.rapacciuolo@unina.it; 6 Ospedale Evangelico Betania, Via Argine 604, 80138 Naples, Italy; 7 Second Division of Cardiology, Pisa University Hospital, 56100 Pisa, Italy; g.zucchelli@ao-pisa.toscana.it (G.Z.); bongiorni@mgbongiorni.it (M.G.B.); 8 Cardiologia, Ospedali Riuniti della Val di Chiana Senesebivio Nottola, 53045 Montepulciano, Italy; ernesto.casorelli@uslsudest.toscana.it; 9 ASST Spedali Civili, Department of Medical and Surgical Specialties, Radiological Sciences and Public Health, University of Brescia, 25123 Brescia, Italy; gianfrancomit@hotmail.com; 10 Klinik für Kardiologie, Angiologie und Intensivmedizin, Deutsches Herzzentrum and Universitätsmedizin Charité Augustenburger Platz 1, 13353 Berlin, Germany; felix.hohendanner@dhzc-charite.de; 11 Department of Precision Medicine, University of Campania “Luigi Vanvitelli”, Via de Crecchio 7, 80138 Napoli, Italy; emanuele.durante@unicampania.it; 12 Unit of Infectious & Transplant Medicine, A.O.R.N. Ospedali dei Colli—Ospedale Monaldi, Piazzale Ettore Ruggieri, 80131 Napoli, Italy; 13 Department of Medical Sciences, University of Turin, 10124 Turin, Italy; veronica.dusi@unito.it; 14 Wiltshire Cardiac Unit, The Great Western Hospitals, Swindon SN5 7YF, UK; p.foley1@nhs.net; 15 Unit of Infectious Diseases, ASST Cremona, 26100 Cremona, Italy; angelo.pan@asst-cremona.it; 16 Cardiology and Intensive Care Unit, Apuane New Hospital, 54100 Massa, Italy; giuseppe.arena@uslnordovest.toscana.it; 17 Department of Cardiology, Liverpool Heart and Chest Hospital, Liverpool L14 3PE, UK; archana.rao@lhch.nhs.uk; 18 Jüdisches Krankenhaus Berlin, Heinz-Galinski-Straße 1, 13347 Berlin, Germany; s_spencker@yahoo.de; 19 Department of Internal Medicine I, TUM University Hospital, Technical University of Munich, 81675 Munich, Germany; 20 Infectious Diseases Clinic, Department of Medicine (DAME), University of Udine, 33100 Udine, Italy; c.tascini@gmail.com; 21 Ospedale Campostaggia, Località Campostaggia 8, 53036 Poggibonsi, Italy; valerio.zaca@uslsudest.toscana.it; 22 Department of Cardiac, Thoracic, Vascular Sciences and Public Health, University of Padova, 35122 Padua, Italy; federico.migliore@unipd.it; 23 Electrophysiology and Cardiac Pacing Unit, Azienda Ospedaliera, Università di Padova, 35128 Padua, Italy; 24 Institute for Skin Integrity, University of Huddersfield, Queensgate, Huddersfield HD1 3DH, UK; ojan.assadian@wienerneustadt.lknoe.at; 25 University Hospital Wiener Neustadt, Corvinusring 3-5, 2700 Wiener Neustadt, Austria; 26 Department of Cardiology, Santa Maria Annunziata Hospital, 50012 Florence, Italy; marzia.giaccardi@uslcentro.toscana.it; 27 Cardiology Unit, Meyer University Hospital IRCCS, Viale Gaetano Pieraccini 24, 50139 Florence, Italy; 28 Department of Internal Medicine and Cardiology, Asklepios Klinikum Schildautal, 38723 Seesen, Germany; 29 Department of Electrophysiology, Heart Center at University of Leipzig, 04289 Leipzig, Germany; kerstin.bode@medizin.uni-leipzig.de

**Keywords:** cardiac implantable electronic device, infection, CIED, pacemaker, cardioverter defibrillator, implantable, antibiotic prophylaxis, biofilm, review, antibiotic-eluting envelope, prevention

## Abstract

**Background:** Cardiac implantable electronic devices (CIEDs) are vital for managing arrhythmias but carry a notable risk of infection, which increases patient morbidity, mortality, and healthcare burden. This review examines current evidence on risk factors and preventive strategies for CIEDI. **Methods:** A structured search was performed in PubMed, Embase, and the Cochrane Library using terms such as “CIED,” “infection,” “pacemaker,” “ICD,” “infection prevention,” “biofilm,” “antibiotic prophylaxis,” and “antibiotic-eluting envelope.” Study selection followed PRISMA guidelines. **Results:** For well-established topics, recommendations are based on high-quality evidence from the literature. In areas with limited CIED-specific data, evidence from related surgical fields was considered, and expert consensus was used to guide recommendations. **Conclusions:** This review offers practical guidance for clinicians on CIED infection prevention, addressing gaps not previously covered in existing guidelines.

## 1. Introduction

Various cardiac arrhythmic conditions necessitate the placement of CIEDs. Nonetheless, this procedure carries inherent risks of adverse events (AEs). These encompass procedure-related issues such as pneumothorax, vascular damage, and hematoma formation, and device-related AEs, such as lead dislodgement or malfunction. Among these, CIED infections stand out as particularly concerning, given their potential to escalate comorbidity, mortality rates, and healthcare resource utilization [1,2,3,4,5,6]. The considerable morbidity, mortality, and strain on healthcare resources attributed to CIED infections in the medical literature prompted the implementation of performance-enhancing measures.

Pathways of CIED Contamination and Infection: CIED hardware contamination often occurs during implantation, likely via contact with the patient’s skin or non-sterile gloves. Colony-forming units (CFUs) introduced at this stage may form biofilms on the device surface. These biofilms can persist silently, explaining why colonization rates exceed clinical infection rates [7,8,9,10]. Once compromised, biofilms may release pathogens, leading to local pocket infections.

In contrast, contamination of transvenous leads typically arises post-implantation through bacteremia or hematogenous seeding [2,3]. Although less frequent, lead-related infections are associated with significantly higher mortality [1,11].

Temporary pacing systems add another risk layer. These external generators connect via percutaneously inserted leads, allowing skin microbiota to colonize the insertion site and transvenous portions of the lead. This risk may increase with factors such as sweating beneath dressings [12]. As a result, temporary pacing prior to permanent implantation elevates the risk of systemic infection.

Understanding these infection routes has led to preventive strategies [3,4,5,13,14].

Yet, despite numerous approaches, evidence remains mixed [15,16,17,18], and healthcare systems face ongoing financial constraints. Hence, a personalized, risk-adapted approach is essential.

Risk stratification based on patient, procedural, and device factors is now central to infection prevention [19,20,21,22]. Several risk calculators exist, with Malagù et al. offering a helpful overview for rapid yet thorough assessment [15,18,23].

However, these tools vary in scope and were often developed outside of dedicated infection studies.

In this comprehensive review, we meticulously assess the risk factors associated with patients, procedures, and devices in the context of CIED infection [Table 1]. We delve into how these factors contribute to the significant increase in infection rates and explore more robust and promising strategies for prevention.

## 2. Materials and Methods

This review was conducted in accordance with the PRISMA 2020 guidelines. The research question was structured using the PICO framework: In adult patients undergoing CIED procedures (Population), do infection prevention strategies (Intervention), compared to standard care or no intervention (Comparator), reduce the incidence of CIED-related infections and associated complications (Outcome)?

A systematic literature search was conducted across PubMed, Embase, and the Cochrane Library from January 2015 to July 2025. The search strategy included a combination of Medical Subject Headings (MeSH) and free-text terms, such as “CIED,” “pacemaker,” “implantable cardioverter-defibrillator,” “infection,” “biofilm,” “infection prevention,” “antibiotic prophylaxis,” “aseptic technique,” “chlorhexidine,” “antisepsis,” “antibiotic-eluting envelope,” and “taurolidine.” Boolean operators (AND, OR) were used to combine terms. Only peer-reviewed articles in English involving adult human participants were included.

Eligible studies were required to report on infection risk factors or infection prevention strategies specific to CIEDs considered “gold standard.” We included randomized controlled trials (RCTs), cohort studies, case–control studies, meta-analyses, systematic reviews, and clinical practice guidelines. Exclusion criteria were non-English articles, animal studies, case reports, editorials, and studies unrelated to infection prevention in CIED recipients. Three reviewers independently screened titles and abstracts, followed by full-text review. Discrepancies were resolved by discussion, resulting in consensus.

The risk of bias was assessed using validated tools according to the study design. RCTs were evaluated using the Cochrane Risk of Bias 2.0 (RoB 2) [24] tool across five domains. Observational studies were assessed with the Robins-I, focusing on participant selection, comparability, and outcome assessment. Systematic reviews and clinical guidelines were assessed using AMSTAR 2 [25] and AGREE II [26], respectively. Each study was independently rated by three reviewers as having low, moderate, or high risk of bias.

Due to methodological heterogeneity among studies, a narrative synthesis was conducted. Findings were organized by thematic categories, including procedural strategies (e.g., skin antisepsis, antibiotic prophylaxis, sterile barriers), device-related innovations (e.g., antibiotic-eluting envelopes, leadless systems, subcutaneous or extravascular devices), and patient-specific risk factors. Quantitative pooling of results was not performed.

In areas lacking direct CIED-specific evidence, such as incision foils, double gloving, pocket irrigation, or fascial plane blocks, data from adjacent surgical fields were considered and the supporting evidence for consensus for recommendation was expanded to the inclusion of case series, narrative reviews and editorials identified in a systematic literature search across PubMed, Embase, and the Cochrane Library as well citation searching, webpages and organizations (i.e., EHRA, AHA, NICE, BHRS) from January 2000–July 2025. Again, all the literature included was assessed with the above-mentioned risk of bias tools [Supplementary Table S1]. Expert consensus was obtained from the faculty (author list) of the second edition of the 360° CIED Infection Congress (Florence, 9 May 2025), representing multidisciplinary agreement on key practices where robust data are currently absent. The PRISMA flow chart can be accessed in the Supplementary Material [Figure S1].

## 3. Results of Literature Search, Assessment, and Consensus

### 3.1. Assessment of CIED-Indication

Preventing CIED-related complications begins with critically assessing the indication for implantation. Many patients may be suitable for deferred placement, particularly as primary prevention indications are being re-evaluated due to advances in medical therapy [27]. Given the potential risks, a substantial portion of CIED therapies may offer limited net benefit [28].

### 3.2. Considerations for Device Selection

For high-risk patients, alternative CIED strategies should be considered. If pacing is not needed, fully subcutaneous systems avoid transvenous leads. When pacing is required, leadless pacemakers offer a promising option. Current technologies include subcutaneous and extravascular ICDs, as well as various leadless pacing systems [29].

### 3.3. Device Programming

Many CIEDs operate with default or non-optimized settings, often programmed by non-specialists [30]. This lack of personalization can shorten battery life, leading to early generator replacements and increased risk of complications, including infections. It also raises concerns about whether current programming practices truly maximize therapeutic benefit while minimizing risk [31].

### 3.4. Generator Exchange

Generator exchanges in CIED patients carry a higher risk of infection due to procedural complexity, biofilm disruption, and impaired local immunity. Each exchange should prompt a reassessment of the CIED indication, including the possibility of deactivation if safe. These procedures are sometimes performed by less experienced operators, increasing the risk of contamination [21,32]. Pathogen colonization is often found on device surfaces at revision [7,9,10]. Biofilms formed during initial implantation can persist for years and may release bacteria when disturbed [33,34,35,36]. Additionally, the fibrous capsule surrounding the device can impair immune response, facilitating bacterial growth [37]. Tissue manipulation and inflammation during exchange further compromise local defenses, enhancing infection risk [38,39,40].

### 3.5. Medication in the Context of CIEDI Prevention

Immunosuppressive medication and oral anticoagulation or dual platelet inhibition are risk factors for CIED-related complications and may interfere with the optimal timing of the CIED procedure [21]. To mitigate the risks associated with these therapies in patients undergoing CIED procedures, a medication-specific algorithm should be followed. For immunosuppressants, elective procedures should be deferred when possible until the patient is on the lowest effective dose, especially for corticosteroids, and infection risk is minimized with close specialist input. For anticoagulants, vitamin K antagonists may be continued if INR is ≤3.0 in high-risk patients, while DOACs should be held on the night and the morning before the intervention based on renal function and bleeding risk, without bridging. Antiplatelet therapy requires careful timing: aspirin alone can be continued, while dual antiplatelet therapy should prompt consideration of postponing the procedure or pausing the P2Y12 inhibitor if outside the high-risk window post-PCI.

### 3.6. Antiseptic Body Bathing

While antiseptic whole-body baths can reduce MRSA colonization, evidence outside specific settings (e.g., ICU patients with CVADs) is inconsistent. Data for CIED patients is lacking. In cases of skin disorders (e.g., colonized diabetic foot ulcers), targeted decolonization based on identified pathogens and susceptibility screening may be considered.

### 3.7. Antiseptic Exit Site Treatment for Temporary Pacing

Post-placement care protocols for central lines can be applied to temporary pacing wires. This includes daily antimicrobial treatment of the lead exit site during dressing changes [41]. Decontamination should use chlorhexidine-gluconate (CHX) in 70% alcohol, allowing time to air dry. If alcohol is contraindicated by the manufacturer, aqueous CHX solutions should be used [41].

### 3.8. Staff Training

Medical staff expertise is critical in preventing CIED infections [21]. No strategy replaces meticulous handling before, during, and after implantation. Training and adherence to evidence-based protocols are essential. In orthopedics, surgeons avoid direct contact with prosthetics to reduce contamination during the critical “race for the surface” phase. Though its impact on infection rates is uncertain, this approach may also benefit CIED procedures and warrants consideration [42].

### 3.9. Procedural Environment and Operating Room Standards

The operating environment is critical for preventing CIED-related infections. Shared operating rooms, especially those used by multiple specialties, increase contamination risk and should be avoided when possible. Optimal suites should maintain minimal bacterial load, ideally with laminar flow and HEPA filtration over the surgical field [43]. This infrastructure reduces airborne pathogen transmission, but strict sterile discipline remains essential. Infection risk increases with unnecessary personnel, uncovered devices, or breaches in gowning protocols. All equipment should be covered with sterile barriers, and room turnover must include thorough cleaning between cases. High-volume centers should carefully manage scheduling and hygiene to prevent cumulative contamination. Where feasible, dedicated rooms for CIED placement are advisable to minimize cross-contamination from other procedures.

### 3.10. Procedural Considerations

Two procedural algorithms (Figures S2 and S3) outline the step-by-step process for CIED placement, incorporating infection prevention and sterile techniques. These algorithms guide clinicians through all infection control steps, from environment selection to proper draping and anesthesia. Evidence shows that such algorithms improve compliance with infection control and reduce infection rates, allowing the interventionalist to focus on procedural details and minimize missed steps [44,45,46].

Since hematoma and postoperative bleeding are among the strongest risk factors for device-related infections, minimizing surgical trauma through efficient, technically precise procedures is critical for infection prevention [47].

### 3.11. Preoperative Antibiotic Administration

Parenteral preoperative antibiotic administration is effectively preventing CIEDI [48]. First-generation cepahlosporins such as Cefazolin is commonly used for prophylaxis in CIED procedures due to its coverage of *Staphylococcus aureus* and *streptococci*, the primary pathogens involved in these infections [49]. Second-generation cephalosporins like cefuroxime provide broader coverage against select Gram-negative organisms, including *Haemophilus influenzae* and certain *Enterobacterales* [50]. However, they do not offer superior efficacy against S. aureus compared to cefazolin and have no advantage in treating β-lactamase-producing staphylococci [49,50]. Neither first- nor second-generation cephalosporins provide coverage against *Pseudomonas aeruginosa* or MRSA, though the former is generally unnecessary, as CIED infections are predominantly caused by S. aureus, *coagulase-negative staphylococci* (typically in generator pocket infections), *streptococci*, and *enterococci* (more commonly in lead-related endocarditis) [51].

Given the poor comparative data, most notably the PADIT trial [18], which showed no benefit of vancomycin over cefazolin prophylaxis even in a high MRSA environment, antibiotic selection should be guided by local microbiological resistance patterns. Regretfully, so far for the prevention of CIED infection possibly caused by MRSA there are no other robust trials other than with vancomycin, which, therefore, remains an alternative in patients with β-lactam allergies or in regions with a high prevalence of MRSA [49]. In high-risk patients, such as those with known colonization with resistant organisms or in institutions at increased MRSA prevalence, targeted susceptibility screening may be considered to optimize prophylactic strategies [2,4,5].

### 3.12. Peri- and Postprocedural Pain Management

Most clinics use tumescent anesthesia with conscious sedation and potentially intravenous sedation for CIED procedures. To minimize skin damage and reduce infection risk, tumescent anesthesia should be administered through a single skin perforation. Ultrasound-guided fascial plane blocks provide effective pain relief and may indirectly lower infection risk by reducing postoperative manipulation of the surgical site [52,53], however direct evidence that fascial-plane blocks reduce surgical-site infection is currently lacking. Combining ropivacaine with clonidine is recommended for prolonged analgesia. Effective pain management supports early mobilization, reducing complications like pneumonia and deep vein thrombosis, which can indirectly contribute to an increased infection risk.

### 3.13. Procedure Packs

Using all-inclusive CIED-placement procedure packs reduces procedural time and infection risk. These packs can be tailored to a center’s needs and should contain all necessary devices for a sterile procedure. Standardized packs streamline training and minimize errors, ensuring consistent practices, such as the preferred drape design for specific CIED placements [54].

### 3.14. Proper Sterile Barrier Conditions

Maximum sterile barrier precautions involve wearing a cap (covering interventionalists’ ears), mask, sterile gown, gloves, and a large sterile drape covering the patient’s body. Using polypropylene gowns and drapes is preferred. These precautions significantly reduce the incidence of CIEDI compared to partial coverage and basic protective measures. Additionally, proper sterile conditions require safe patient restraints, especially if conscious sedation is used, to prevent irregular movements during the procedure.

### 3.15. Hand Hygiene

Hand hygiene is critical for infection prevention. CFUs can multiply on gloved hands, making it essential to wash hands and forearms with soap and water when visibly soiled, followed by alcohol-based sanitizer. If hands are not soiled, alcohol-based sanitizer can be used between procedures. Chlorhexidine-based soaps may cause allergic reactions in 20% of healthcare workers, while alcohol-based sanitizers are less irritating, quicker to use, more cost-effective, and reduce water usage. They also maintain long-term efficacy and prevent bacterial resistance [55,56,57,58,59,60,61]. Hand hygiene is also essential during handling of wound dressing.

### 3.16. Skin Antisepsis

Skin antisepsis with 2% chlorhexidine gluconate and ethanol has shown inconsistent superiority over PVP-I. Alcoholic CHX preparations are preferred for their faster drying time, prolonged activity, and broader antimicrobial spectrum. If alcoholic CHX is unavailable, olanexidine should be preferred over PVP-I [62].

### 3.17. Incision Drapes

Use of iodine-impregnated incision drapes in CIED procedures, such as pacemaker or ICD placement or revision, has been associated with reduced surgical site infection risk due to their sustained antiseptic activity. Scientific evidence supports their efficacy: a large prospective study by Golian M. et al. in patients undergoing CIED placement procedures demonstrated that the use of iodophor-impregnated drapes significantly reduced postoperative infections compared to standard sterile drapes alone [63]. These findings are supported by a recently published RCT [64]. Iodine, being a broad-spectrum antimicrobial agent, provides continuous antisepsis at the incision site during the procedure, effectively decreasing skin flora contamination. Given the high morbidity associated with CIED infections, the integration of antiseptic barriers like iodine-impregnated drapes represents a valuable preventive strategy.

### 3.18. CIED Placement

Creation of the pocket for placement of the CIED generator may be performed using blunt dissection, scissors, electrocautery, or a Pulsed Electron Avalanche Knife (PEAK). The PEAK device is particularly advantageous when re-entering a pre-existing pocket, as it minimizes the risk of mechanical or thermal damage to the implanted hardware [65].

Adverse events are less frequent with submuscular placement of CIEDs compared to subcutaneous placement, and for S-ICDs, an intermuscular position (M. Serratus and M. Lat. Dorsi) is recommended. Most adverse events are due to surgical errors during initial placement. Vascular access for transvenous leads should be prepared with cephalic vein access, as it reduces complications like lead dislodgement and pneumothorax. Site selection should consider anatomy, patient preferences, comorbidities, and prior hardware, with preoperative imaging (ultrasound or venography) recommended. Intraoperative changes to the implantation site should be avoided to maintain aseptic conditions [66,67].

### 3.19. Antibiotic Eluting Envelope

While coated CVADs effectively reduce bloodstream infections, attempts to coat cardiac CIEDs have failed to prevent infections. As an alternative, antibiotic-eluting mesh envelopes have been developed. These absorbable envelopes, containing minocycline and rifampin, release antibiotics over time to protect against bacterial colonisation and effectively reduce CIED infections, particularly in high-risk patients, such as those undergoing generator changes. [68,69,70,71,72,73] The antibiotic-eluting envelope, while effective, incurs additional costs and requires procedural adjustments, including enlarging the fibrous pocket. This incision triggers a protective immune response, potentially aiding infection prevention and tissue healing. The deployment algorithm is shown in Figure S2.

### 3.20. Extracellular Matrix Envelope

Biological small bowel submucosal envelopes infused with extracellular matrix reduce fibrotic lead entrapments, facilitate easier manipulation of the generator and lead, and result in thinner tissue capsules during reoperation. However, clinical experience with these envelopes in existing fibrotic capsules is limited. Some envelopes are impregnated with antiseptics or antibiotics, such as gentamicin, which has been shown to reduce infection risk by threefold in high-risk patients [71,74,75].

### 3.21. Antibiotic Pocket Irrigation

Antibiotics act as substrates for bacterial metabolism, either killing bacteria (bactericidal) or inhibiting their growth (bacteriostatic). They are only effective when bacteria are actively multiplying and present at minimal inhibitory concentrations over time. Flushing antibiotics into CIED pockets does not effectively prevent bacterial growth. A large study on parenteral antibiotics combined with topical application in CIED pockets found no significant benefit, so local antibiotic irrigation is discouraged [3,4,5,18].

### 3.22. Antimicrobial Compound Pocket Irrigation

Antimicrobial agents for pocket irrigation act differently than antibiotics, quickly destroying various germs (bacteria, viruses, fungi) and preventing CIED colonization and infection. While used globally with various antiseptics, one medical device is certified for use during CIED procedures, providing safety data for its intended use. Below are disinfectants and antiseptics used during CIED placements:

Licensed use:

Taurolidine-containing antimicrobial adjunct: Taurolidine, derived from taurine, is a key antimicrobial agent used in CIED procedures and central venous access device locking solutions. A deployment algorithm for taurolidine is shown in Figure S3. SOPs can be downloaded from etpr.eu and are available in the Supplementary Material [14,76,77,78,79,80,81,82,83].

Off-license use:

Ethanol-based antiseptics are commonly used as deep incisional wound irrigants in various medical settings. One significant concern with ethanol-based antiseptics is their potential to denature proteins [84]. Protein denaturation during wound irrigation can hinder natural healing [85], delay wound closure, and promote bacterial colonization and biofilm formation, particularly by S. aureus [36,85,86,87,88].

Chlorhexidine Gluconate 2%—aqueous: due to its ability to induce allergic reaction and chemical burns, as well as systemic adsorption it needs to be removed with saline or Ringer’s solution after deep incisional wound irrigation [89]. These safety issues prevent its adoption in a wider context of CIED procedures.

Chlorhexidine Gluconate 2–70% ethanol: similarly, the ethanolic sibling’s employment during CIED procedures must be discouraged due to its side effects [89] even though it has been hinted at reduced CIEDI when used in one study, where it was subsequently removed by saline irrigation after its employment [90].

Hydrogen peroxide: has been utilized for decades as an antiseptic agent due to its ability to kill bacteria, viruses, and fungi. However, its effectiveness in preventing surgical site infections remains inconsistent [77,91,92]. We advise against using hydrogen peroxide for wound irrigation due to its cytotoxic effects, which can damage tissue, delay healing, and harm CIED hardware [93,94,95,96,97,98,99].

Hypochlorous acid: Despite its antimicrobial efficacy, its corrosive nature may compromise CIED components, potentially causing malfunction or premature failure. Caution is needed to ensure optimal device performance [94].

Octenidine: must be avoided in deep incisional wounds to prevent CIED infection, as it may cause tissue irritation, necrosis, and delay healing [94,100,101].

PVP-iodine: use should be avoided to prevent CIED infection due to its potential cytotoxicity and adverse effects on wound healing. PVP iodine can cause tissue irritation and allergic reactions, leading to delayed wound healing and increased risk of infection. [102] Additionally, most PVP iodine solutions contain ethanol [103].

Polyhexanide: Polyhexamethylene biguanide (PHMB) is a disinfectant used in wound care but may cause tissue irritation, allergic reactions, and delay healing. It can also disrupt CIED functionality and contribute to bacterial resistance, underscoring the need for proper disinfection and ongoing resistance monitoring [89,104].

### 3.23. Suture Materials and Techniques

In the context of CIED surgery, the choice of suture materials and techniques is crucial for achieving optimal wound closure and reducing the risk of complications such as infection and wound dehiscence.

Definitions of Suture Materials:

Absorbable vs. Non-absorbable: Absorbable sutures, such as polyglactin or polydioxanone, gradually degrade over time and are eventually absorbed by the body. Non-absorbable sutures, such as polypropylene or nylon, remain in the body indefinitely unless removed.

Monofilament vs. Multifilament: Monofilament sutures consist of a single strand of material, offering smooth passage through tissues and reduced risk of bacterial adherence. Multifilament sutures are composed of multiple strands twisted or braided together, providing greater flexibility and knot security but potentially higher risk of infection due to increased surface area.

Suture Techniques:

Interrupted Sutures: Interrupted sutures, placed at regular intervals, ensure precise wound edge approximation and tension adjustment. They are preferred for contaminated or high-tension wounds where alignment is critical [105].

Continuous Sutures: Continuous sutures provide quick, uniform wound closure but may increase the risk of dehiscence if a section fails [105].

Subcuticular Sutures: Subcuticular sutures offer a cosmetically pleasing closure and reduce the risk of irritation or infection, making them ideal for CIED surgery [106].

Figure-of-Eight Sutures: Figure-of-eight sutures evenly distribute tension across high-stress areas, reducing the risk of tissue ischemia or necrosis [107].

Infection rates were evenly distributed among interrupted and continuous suture techniques in a recent review [105].

### 3.24. Considerations in Respect to Suture Materials and Techniques

Infection Risk: To minimize infection risk, absorbable, monofilament sutures are preferred for superficial intracutaneous closure. Interrupted sutures should be avoided. Subcutaneous tissue should be secured with resorbable sutures, while non-resorbable sutures should be used for sleeve fixation to prevent lead displacement [106,108].

Tissue Handling: Gentle tissue handling during suturing is crucial to minimize trauma and ischemia, promoting wound healing and reducing complications. Suture materials and techniques in CIED surgery should prioritize healing, infection prevention, and patient safety, while considering individual patient factors and surgeon expertise.

Tissue adhesive is a clinically effective alternative to monofilament sutures for skin closure, offering comparable wound healing outcomes. Although it is generally more costly, it may reduce procedure time and improve patient comfort. Adequate hemostasis is essential prior to application to ensure optimal adhesion and wound approximation [109].

### 3.25. Wound Dressings

Various pressure devices are available for use after CIED generator implantation to reduce complications like hematoma or wound dehiscence. However, there is limited evidence on their effectiveness, making it difficult to recommend their use. As a more cost-effective alternative, stretch bandages can provide similar benefits with adjustable compression [110].

### 3.26. Surveillance

CIEDI rates and surveillance methods affect data collection and interpretation [111,112]. Diagnosing CIEDI can be subjective, as medical professionals may interpret criteria differently based on clinical experience, biases, and external influences. While guidelines aim for standardization, human factors introduce variability, highlighting the need for collaborative approaches. In regions with public reporting and financial penalties, diagnostic definitions may shift, making it difficult to distinguish between actual clinical improvements and changes in reporting practices.

### 3.27. Future Perspective

CIEDI rates are influenced by factors such as patient comorbidities, device complexity, and antibiotic resistance. To address these challenges, adaptable prevention strategies are essential. While high-income countries often implement advanced measures, globally accessible solutions, such as simplified algorithms, remain crucial for scalability and resilience across diverse healthcare settings.

## 4. Summary

Preventing CIED complications requires rigorous measures, including staff training, evidence-based algorithms, and continuous monitoring. Re-evaluating the need for CIED placement and considering alternatives like subcutaneous ICDs or leadless pacemakers can reduce infection risks. Infection prevention involves standardized practices, such as site selection, pre-operative antibiotics, strict sterile techniques, and effective anaesthesia. Additional measures, like MRSA decolonisation, care algorithms for pacing wires, taurolidine-containing solution adjuncts, and antibiotic-eluting mesh envelopes, can further reduce CIEDI risks.

## Figures and Tables

**Table 1:** Pooled effect estimates for potential risk factors predisposing to CIED infection. Adapted from Polyzos et al. [21].

Factor	Statistical Method	Pooled Estimate	*p* Value
**Host-related risks**			
age	weighted mean difference	−1.27 [−3.08, 0.55]	0.17
male gender	OR	1.12 [0.89, 1.41]	0.35
body mass index (BMI) ≥ 25	OR	1.04 [0.63, 1.72]	0.87
body weight	weighted mean difference	−2.69 [−6.69, 1.31]	0.19
American Society of Anesthesiologists (ASA) risk classification ≥ 3	OR	0.93 [0.24, 3.58]	0.91
smoking	OR	0.48 [0.11, 2.08]	0.32
diabetes mellitus	OR	2.08 [1.62, 2.67]	<0.000001
renal insufficiency (glomerular filtration rate < 60 mL)	OR	3.02 [1.38, 6.64]	0.006
end-stage renal disease (glomerular filtration rate < 15 mL)	OR	8.73 [3.42, 22.31]	0.00001
serum Creatinine	weighted mean difference	12.78 [−9.78, 35.33]	0.27
cirrhosis	OR	1.94 [0.28, 13.53]	0.51
atrial fibrillation	OR	1.12 [0.63, 1.98]	0.69
coronary vessel disease	OR	1.26 [0.92, 1.73]	0.15
prior myocardial infarction	OR	1.08 [0.41, 2.89]	0.87
coronary artery bypass graft	OR	0.87 [0.54, 1.40]	0.56
chronic heart failure	OR	1.65 [1.14, 2.39]	0.008
New York Heart Association (NYHA) class ≥ 2	OR	2.47 [1.24, 4.91]	0.01
left ventricular ejection fraction	weighted mean difference	−0.78 [−3.32, 1.76]	0.55
prosthetic valve replacement therapy	OR	1.42 [0.72, 2.81]	0.31
peripheral vessel disease	OR	0.88 [0.31, 2.50]	0.81
cerebrovascular disease	OR	1.07 [0.30, 3.79]	0.91
chronic obstructive pulmonary disease	OR	2.95 [1.78, 4.90]	0.00003
malignancy/neoplasia	OR	2.23 [1.26, 3.95]	0.006
autoimmune disease	OR	1.44 [0.42, 4.90]	0.56
skin disorders	OR	2.46 [1.04, 5.80]	0.04
fever within 24 h prior to implantation	OR	4.27 [1.13, 16.12]	0.03
oral anticoagulants	OR	1.59 [1.01, 2.48]	0.04
platelet inhibition	OR	1.37 [0.83, 2.28]	0.22
Heparin bridging	OR	1.87 [1.03, 3.41]	0.04
Corticosteroid use	OR	3.44 [1.62, 7.32]	0.001
immunosuppressive drug use (other than corticosteroids)	OR	1.85 [0.63, 5.46]	0.27
permanent central venous access device	OR	5.74 [0.94, 34.93]	0.06
prior device infection	OR	7.84 [1.94, 31.60]	0.004
**Procedure-related risks**			
antibiotic prophylaxis	OR	0.32 [0.18, 0.55]	0.00005
device replacement/revision/upgrade	OR	1.98 [1.46, 2.70]	0.00001
generator change	OR	1.74 [1.22, 2.49]	0.002
lead dislodgement/repositioning	OR	6.37 [2.93, 13.82]	0.000003
hematoma formation	OR	8.46 [4.01, 17.86]	<0.000001
temporary pacing	OR	2.31 [1.36, 3.92]	0.002
procedure duration	weighted mean difference	9.89 [0.52, 19.25]	0.04
inexperienced operator	OR	2.85 [1.23, 6.58]	0.01
**Device-related risks**			
implantable cardioverter defibrillator (ICD) device	OR	1.19 [0.84, 1.68]	0.32
cardiac resynchronization (CRT) device	OR	1.92 [0.90, 4.10]	0.09
dual-chamber device (i.e., pacemaker with atrial and ventricular lead)	OR	1.45 [1.02, 2.05]	0.04
≥2 leads	OR	2.02 [1.11, 3.69]	0.02
abdominal generator pocket	OR	4.01 [2.48, 6.49]	<0.000001
epicardial leads	OR	8.09 [3.46, 18.92]	0.000001
abandoned leads	OR	1.82 [0.86, 3.83]	0.12

## Data Availability

Data analysis will be provided upon reasonable request to the corresponding author.

## Supplementary Materials

The following supporting information can be downloaded at https://www.mdpi.com/article/10.3390/jcdd12100406/s1, Figure S1: PRISMA flowchart showing the selection process of studies included in the review on CIED infection prevention.; Figure S2: Procedural algorithm outlining the use of an antibiotic envelope in high-risk CIED procedures; Figure S3: Stepwise procedural algorithm demonstrating the adjunctive application of Taurolidine for infection prevention during CIED procedures; Table S1: Risk of Bias Assessment of Literature Included in This Review.

## Abbreviations

The following abbreviations are used in this manuscript:

CIED	Cardiac Implantable Electronic Device
CIEDI	Cardiac Implantable Electronic Device Infection
ICD	Implantable Cardioverter-Defibrillator
CRT	Cardiac Resynchronization Therapy
PRISMA	Preferred Reporting Items for Systematic Reviews and Meta-Analyses
RCT	Randomized Controlled Trial
RoB 2	Risk of Bias 2.0 Tool
NOS	Newcastle-Ottawa Scale
AMSTAR 2	A MeaSurement Tool to Assess Systematic Reviews 2
AGREE II	Appraisal of Guidelines for Research and Evaluation II
MeSH	Medical Subject Headings
OR	Odds Ratio
RR	Relative Risk
