# Supplementary material for: Addressing Complications in Cardiac Implantable Electronic Devices: A Guideline to Prevention of CIED Infection
Source: J Cardiovasc Dev Dis. 2025 Oct 13;12(10):406. doi: 10.3390/jcdd12100406 (PMC12564914; doi:10.3390/jcdd12100406)
Supplement: Supplementary file 1 [file jcdd-12-00406-s001.zip › Figure_S1_PRISMA_flow_diagram.pdf]

PRISMA Flow Diagram

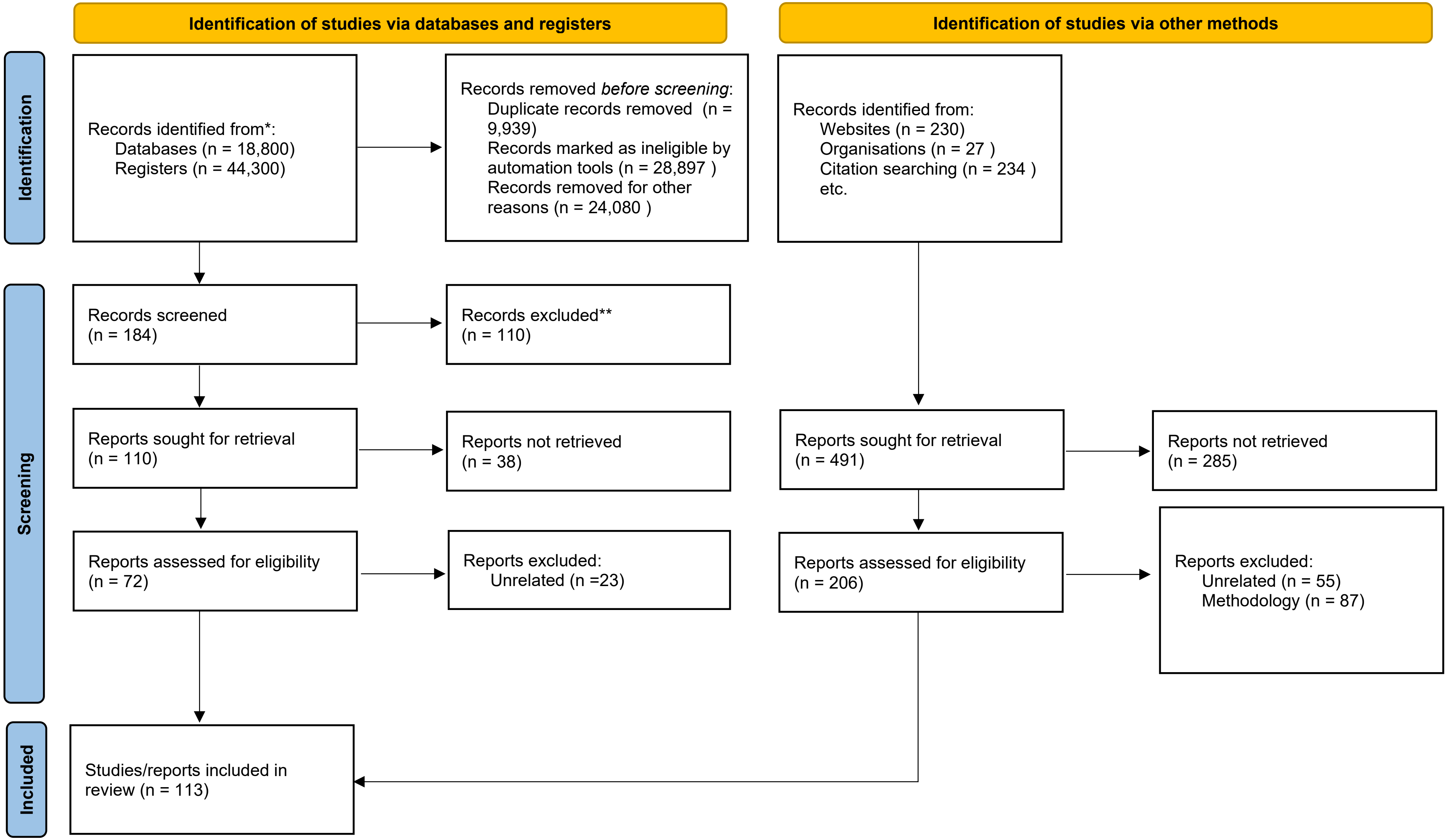

Source: Page MJ, et al. BMJ 2021;372:n71. doi: 10.1136/bmj.n71.

This work is licensed under CC BY 4.0. To view a copy of this license, visit https://creativecommons.org/licenses/by/4.0/
