# Supplementary material for: Addressing Complications in Cardiac Implantable Electronic Devices: A Guideline to Prevention of CIED Infection
Source: J Cardiovasc Dev Dis. 2025 Oct 13;12(10):406. doi: 10.3390/jcdd12100406 (PMC12564914; doi:10.3390/jcdd12100406)
Supplement: Supplementary file 1 [file jcdd-12-00406-s001.zip › Table_S1_suppl.pdf]

Table S1. Risk of Bias Assessment of Selected Studies on Cardiac Implantable Electronic Device Interventions to Prevent or Mitigate Infections.

**Ref**	**Study Type**	**Tool Used**	**Risk of Bias**	**Notes**
1. Pokorney et al. (2023) – JAMA Cardiol	Retrospective cohort	ROBINS-I	**Moderate**	Large registry study, adjusted for confounders, but potential residual confounding and selection bias exist.
2. Fowler et al. (2023) – Clin Infect Dis	Diagnostic criteria consensus	N/A	N/A	Methodological paper updating diagnostic criteria. No direct intervention/outcome data.
3. Baddour et al. (2024) – Circulation	Scientific statement / expert consensus	AGREE II	**High credibility**	Authoritative guideline; not subject to classic RoB but based on best available evidence.
4. Blomstrom-Lundqvist et al. (2020) – Eur Heart J	Consensus guideline	AGREE II	**High credibility**	Multisociety consensus document, high methodological quality.
5. Traykov et al. (2019) – Europace	International survey	N/A	**Low - Moderate**	Observational self-reported data; risk of response bias.
6. Sohail et al. (2016) – Circ Arrhythm Electrophysiol	Retrospective database analysis	ROBINS-I	**Moderate - High**	Claims data prone to coding errors; adjustment may not fully eliminate confounding.
7. Inacio et al. (2015) – Int J Infect Dis	Cross-sectional lab study	ROBINS-I	**Moderate**	Focus on diagnostic yield of sonication; no intervention. Bias from selection and lack of control group.
8. Chu et al. (2014) – Biomed Res Int	Observational with lab/genetic component	ROBINS-I	**Moderate**	Small sample; uses genetic tools but lacks randomization or control group.
9. Okada et al. (2013) – Eur Heart J (abstract)	Observational (abstract only)	N/A	**High**	not peer-reviewed full article; limited data and unclear methods.
10. Kleemann et al. (2010) – Europace	Prospective observational	ROBINS-I	**Moderate**	Well-structured but still non-randomized with potential selection bias.

Table S1. Risk of Bias Assessment of Selected Studies on Cardiac Implantable Electronic Device Interventions to Prevent or Mitigate Infections.

Ref	Study	Study Type	Tool	Risk of Bias	Notes
**11**	Sohail et al. (2015)	Retrospective cohort	ROBINS-I	**Moderate**	Solid design with long-term follow-up, but subject to confounding and selection bias.
**12**	Frausing et al. (2022)	Nationwide cohort (Denmark)	ROBINS-I	**Low - moderate**	High-quality registry; low selection bias but no randomization.
**13**	Blomstrom-Lundqvist & Ostrowska (2021)	Guideline/review	AGREE II	**High credibility**	Based on expert consensus and supported by strong evidence.
**14**	Foley et al. (2024)	National standards document	AGREE II	**High credibility**	Practice standard; not a study, but authoritative.
**15**	Maclean et al. (2024)	Prognostic tool development/validation	ROBINS-I	**Moderate**	Good methodology and external validation; some modeling assumptions.
**16**	Chaudhry et al. (2022)	Observational (high-risk patients)	ROBINS-I	**Moderate**	Cohort comparison without randomization; risk of confounding.
**17**	Biffi (2019)	Commentary/editorial	N/A	**N/A**	Opinion-based, not data-driven.
**18**	Krahn et al. (2018) – PADIT Trial	RCT	Cochrane RoB 2	**Low**	Large, multicenter RCT; low risk of bias.
**19**	Olsen et al. (2022)	Nationwide cohort (Denmark)	ROBINS-I	**Low**	Comprehensive dataset with minimal missing data; well-conducted.
**20**	Olsen et al. (2019)	Retrospective cohort (national registry)	ROBINS-I	**Moderate**	Longitudinal data; typical limitations of retrospective design.

Table S1. Risk of Bias Assessment of Selected Studies on Cardiac Implantable Electronic Device Interventions to Prevent or Mitigate Infections.

Ref	Study	Study Type	Tool	Risk of Bias	Notes
**21**	Polyzos et al. (2015)	Systematic review & meta-analysis	AMSTAR 2	**Moderate - High**	Well-structured, but likely included heterogeneous and lower-quality studies; some risk of bias in included studies.
**22**	Ishiguchi et al. (2020)	Single-center observational (20 years)	ROBINS-I	**Moderate - High**	Long duration adds value, but single-center limits generalizability; selection and information bias possible.
**23**	Malagù et al. (2022)	Comparative risk score analysis	ROBINS-I	**Moderate**	No RCT data; relies on retrospective modeling. Useful but exploratory.
**24**	Martimbianco et al. (2023)	Meta-research (Cochrane review audit)	N/A	**N/A**	Methodology critique, not a clinical study. No patient data or outcomes involved.
**25**	Shea et al. (2017)	AMSTAR 2 tool description	N/A	**N/A**	Tool development paper, not a clinical study.
**26**	Brouwers et al. (2010)	AGREE II guideline development	N/A	**N/A**	Methodology framework paper.
**27**	Dagres et al. (2020) – PROFID project	Study protocol/project description	N/A	**N/A**	Descriptive only; no patient-level outcome data yet.
**28**	Döring et al. (2020)	Retrospective observational	ROBINS-I	**Moderate**	Practical study on extraction and reimplantation, but not randomized; subject to bias.
**29**	Al-Khatib (2024)	Narrative review (NEJM)	N/A	**N/A**	High-level summary from expert, not original data.
**30**	Üreyen et al. (2017)	Opinion/editorial	N/A	**N/A**	Viewpoint article on follow-up responsibility; not empirical research.

Table S1. Risk of Bias Assessment of Selected Studies on Cardiac Implantable Electronic Device Interventions to Prevent or Mitigate Infections.

Ref	Study	Study Type	Tool	Risk of Bias	Notes
**31**	Lau, E.W. (2017)	Narrative review	N/A	**N/A**	Descriptive review of CIED longevity technologies; lacks systematic methodology.
**32**	Boriani et al. (2022)	Nationwide registry cohort	ROBINS-I	**Moderate - High**	Observational data with potential confounding and registry limitations.
**33**	Qu, Peleg & McGiffin (2021)	Narrative review	N/A	**N/A**	Clinical review on VAD-related infections; not a primary study.
**34**	Colombari et al. (2021)	In-vitro experimental study	N/A	**Moderate**	Controlled experimental work; unclear blinding/replication.
**35**	Masters et al. (2019)	Narrative review	N/A	**N/A**	Theoretical review on bone infection and biofilm; no empirical bias assessment.
**36**	Luther et al. (2018)	Observational genetic study	ROBINS-I	**Moderate - High**	Observational design with possible confounding and selection bias.
**37**	Sharma et al. (2023)	Narrative review	N/A	**N/A**	Comprehensive overview of microbial biofilms; descriptive synthesis only.
**38**	Wolfram et al. (2004)	Observational histological study	Newcastle–Ottawa	**Moderate**	Retrospective tissue analysis; unclear blinding of assessors.
**39**	Ludwig et al. (2018)	Retrospective claims analysis	ROBINS-I	**High**	Administrative data; coding errors and confounding possible.
**40**	Lakkireddy et al. (2015)	Randomized controlled trial	Cochrane RoB 2	**Low - Moderate**	Prospective RCT; some uncertainty about blinding and allocation concealment.
**41**	NICE (2017)	Clinical guideline	AGREE II	**High credibility**	Well-established national policy; strong methodological rigor.

Table S1. Risk of Bias Assessment of Selected Studies on Cardiac Implantable Electronic Device Interventions to Prevent or Mitigate Infections.

	Ref	Study	Study Type	Tool	Risk of Bias
**42**	Gristina et al. (1988)	Narrative review	N/A	**N/A**	Foundational theoretical work; not empirical research.
**43**	Carlà et al. (2025)	Retrospective observational study	ROBINS-I	**Moderate - High**	Single-center 3-year data; potential selection and confounding bias.
**44**	Meddings & Saint (2011)	Narrative review	N/A	**N/A**	Opinion-based educational commentary; no primary data.
**45**	Manojlovich et al. (2016)	Observational study	ROBINS-I	**Moderate**	Real-world data on catheter practices; some observer and confounding bias.
**46**	Lo et al. (2014)	Clinical guideline	AGREE II	**High credibility**	Evidence-based CAUTI prevention update; transparent development process.
**47**	Mehta et al. (2021)	Narrative review	N/A	**N/A**	Review on hematoma prevention in CIED implantation; descriptive synthesis.
**48**	Da Costa et al. (1998)	Meta-analysis	AMSTAR 2	**Moderate**	Systematic synthesis of RCTs; older data may limit comprehensiveness.
**49**	Bratzler et al. (2013)	Clinical practice guideline	AGREE II	**High credibility**	Multisociety surgical prophylaxis guideline; robust evidence grading.
**50**	Leekha et al. (2011)	Narrative review	N/A	**N/A**	Educational overview of antimicrobial principles; not empirical research.
**51**	Sohail et al. (2007)	Retrospective cohort study	ROBINS-I	**Moderate - High**	Real-world data on pacemaker/ICD infections; risk of confounding and selection bias.
**52**	Chin et al. (2021)	Anatomical review	N/A	**N/A**	Anatomical insights for regional blocks; no clinical outcomes.

Table S1. Risk of Bias Assessment of Selected Studies on Cardiac Implantable Electronic Device Interventions to Prevent or Mitigate Infections.

	Ref	Study	Study Type	Tool	Risk of Bias
**53**	Capuano et al. (2024)	Narrative review	N/A	**N/A**	Summarizes fascial blocks in cardiothoracic surgery; not a primary study.
**54**	Calò et al. (2023)	Systematic review	AMSTAR 2	**Moderate**	Attempts to consolidate procedural factors; quality dependent on included studies.
**55**	Stang et al. (2022)	Historical perspective (epidemiology)	N/A	**N/A**	Methodological analysis of Semmelweis' work; not clinical research.
**56**	Camarasa (2001)	Book chapter	N/A	**N/A**	Educational content in dermatology reference; no original data.
**57**	WHO (2009)	Global guideline	AGREE II	**High credibility**	Foundational WHO hand hygiene guidance; widely adopted.
**58**	FDA (2022)	Regulatory safety alert	N/A	**N/A**	Safety communication on CHG; not a research study.
**59**	Widmer (2013)	Narrative review	N/A	**N/A**	Review on surgical hand hygiene techniques; no original study design.

Table S1. Risk of Bias Assessment of Selected Studies on Cardiac Implantable Electronic Device Interventions to Prevent or Mitigate Infections.

Ref	Study	Study Type	Tool	Risk of Bias	Notes
**60**	Tavolacci et al. (2006)	Comparative cost-effectiveness study	ROBINS-I	**Moderate**	Small sample and limited follow-up, but relevant for hygiene strategy comparison.
**61**	Kampf & Ostermeyer (2005)	Controlled trial (hand rub efficacy)	Cochrane RoB 2	**Low - Moderate**	Experimental setup; possibly limited blinding and external validity.
**62**	Jalalzadeh et al. (2022)	Systematic review & network meta-analysis	AMSTAR 2	**Moderate**	Consistent methodology, registered with PROSPERO, GRADE applied; some risk of bias and concerns in several RCTs, moderate heterogeneity, limited to one RCT for olanexidine; overall solid but with limitations.
**63**	Golian et al. (2024)	Large prospective cohort	ROBINS-I	**Moderate**	Prospective and robust, but non-randomized; possible selection bias.
**64**	Aydin et al. (2025)	Randomized clinical trial	Cochrane RoB 2	**Low**	Well-designed single-center RCT with adequate randomization, blinding of outcome assessment, and prespecified protocol. Some limitations due to single-center design and small number of infections observed.
**65**	Peprah et al. (2019)	Narrative review	N/A	**Moderate**	Review of PEAK PlasmaBlade versus traditional electrocautery; based on secondary data and modeling. Not primary clinical research; conclusions limited by quality of included evidence and assumptions in cost-effectiveness analysis
**66**	Gallik et al. (1996)	Retrospective comparative study	ROBINS-I	**Moderate - High**	Outdated methods and potential bias due to lack of randomization.
**67**	Droghetti et al. (2022)	Case series (conservative technique)	N/A	**High**	Very small sample; lacks controls; primarily descriptive.

Table S1. Risk of Bias Assessment of Selected Studies on Cardiac Implantable Electronic Device Interventions to Prevent or Mitigate Infections.

Ref	Study	Study Type	Tool	Risk of Bias	Notes
**68**	Ziacchi et al. (2023) – REINFORCE Project	Real-world registry	ROBINS-I	**Moderate**	Observational registry data; risk of unmeasured confounding.
**69**	Mittal et al. (2020) – WRAP-IT Long-term	Randomized controlled trial (extension)	Cochrane RoB 2	**Low**	High-quality follow-up of WRAP-IT; minimal bias in outcome measurement.
70	Tarakji et al. (2019) – WRAP-IT Trial	Multicenter RCT	Cochrane RoB 2	**Low**	Landmark trial; rigorous design and execution.
**71**	Woodard et al. (2022)	Retrospective observational	ROBINS-I	**Moderate**	Outcomes by patient risk profile; possible coding or selection bias.
**72**	Khalil et al. (2020)	Prospective case series	ROBINS-I	**Moderate - serious**	Non-randomized, high-risk population; comprehensive intervention but lacks control group.
**73**	Henrikson et al. (2017) – Citadel/Centurion	Prospective observational	ROBINS-I	**Moderate**	Sponsored study, but large sample and prospective design support validity.
**74**	Deering et al. (2022)	Survey-based preference study	N/A	**High (reporting bias)**	Physician-reported preferences are subjective; not clinical outcomes.
**75**	Sohail et al. (2020)	Preclinical animal model	SYRCLE RoB	**Moderate**	Good experimental control; limited generalizability to human outcomes.
**76**	Vonthein et al. (2023) – TauroPace Registry Protocol	Study protocol	N/A	**N/A**	Describes methods; bias not applicable until study results are published.

Table S1. Risk of Bias Assessment of Selected Studies on Cardiac Implantable Electronic Device Interventions to Prevent or Mitigate Infections.

Ref	Study	Study Type	Tool	Risk of Bias	Notes
**77**	Borov et al. (2023)	Retrospective observational	ROBINS-I	**Moderate**	Promising outcomes with Taurolidine, but design limits causal inference.
**78**	Salucci et al. (2024)	Observational abstract/poster	ROBINS-I	**Moderate - High**	Limited data available; high risk of publication bias and reporting bias.
**79**	De Vivo et al. (n/a)	Case report	N/A	**High**	Single COVID-related case; not generalizable.
**80**	Giudice et al. (2023)	Case report	N/A	**High**	Demonstrates Taurolidine use; limited evidence level.
**81**	Borov et al. (2022)	Case report	N/A	**High**	Descriptive account of salvage treatment; anecdotal evidence.
**82**	Giaccardi et al. (2022)	Case report	N/A	**High**	Frail patient salvage case; useful anecdote but not generalizable.
**83**	Weichsel et al. (2022)	Case series (pediatrics)	ROBINS-I	**Moderate - High**	Small sample; specific to VAD patients; lacks comparator.
**84**	Sweetman (2009)	Drug reference (book)	N/A	**N/A**	Drug compendium; not original research.
**85**	Keast & Orsted (1998)	Educational review	N/A	**N/A**	Wound care principles; non-empirical content.
**86**	Redelman et al. (2012)	In vitro study	N/A	**Moderate**	Laboratory findings on alcohol-induced biofilm; indirect clinical relevance.
**87**	Luther et al. (2015)	In vitro study	N/A	**Moderate**	Confirms alcohol exposure and biofilm formation; non-clinical.
**88**	Sohail et al. (2021) – WRAP-IT Microbiology Analysis	Substudy of RCT	Cochrane RoB 2	**Low**	High-quality post hoc analysis; consistent with parent WRAP-IT trial.
**89**	Schunter et al. (2017)	Case report	N/A	**High**	Describes rare anaphylaxis; not generalizable, but valuable safety alert.

Table S1. Risk of Bias Assessment of Selected Studies on Cardiac Implantable Electronic Device Interventions to Prevent or Mitigate Infections.

Ref	Study	Study Type	Tool	Risk of Bias	Notes
**90**	Diaz et al. (2023)	Prospective observational	ROBINS-I	**Moderate**	Evaluates CHG pocket irrigation; no randomization but clinically relevant data.
**91**	Apel et al. (2018)	Retrospective observational	ROBINS-I	**Moderate - High**	Examines H_2O_2 application; potential for confounding and incomplete data.
**92**	Caid et al. (2022)	Narrative review	N/A	**N/A**	General summary of irrigation solutions; no primary data.
**93**	Kiran et al. (2018)	Case report	N/A	**High**	Single case of air embolism; limited generalizability.
**94**	Kramer et al. (2017)	Expert consensus	AGREE II	**High credibility**	Moderate methodological rigor; expert-based guidance without formal systematic review.
**95**	Yang et al. (2016)	Narrative review	N/A	**Moderate**	Raises theoretical risk of H_2O_2; lacks systematic evidence.
**96**	Zhang et al. (2015)	Case report	N/A	**High**	Rare case of cerebral embolism from H_2O_2; low generalizability.
**97**	Lake (2009)	Case commentary	N/A	**High**	Brief correspondence on embolism; anecdotal evidence only.
**98**	Guerin et al. (2006)	Hypothesis article	N/A	**High**	Speculative, no empirical study; raises hypothesis about aseptic loosening.
**99**	Cannon et al. (2003)	Case report	N/A	**High**	Pediatric neurotoxicity from H_2O_2; important but single case.
**100**	Seegräber et al. (2017)	Case report	N/A	**High**	Local skin reaction post-Octenidine; low generalizability.
**101**	Högele & Neu (2011)	Case report	N/A	**High**	Describes adverse outcome post-wound irrigation; anecdotal.
**102**	Sammartino et al. (2012)	Case report	N/A	**High**	Post-extraction iodine reaction; not generalizable.
**103**	Takeda et al. (2024)	Randomized controlled trial (RCT)	Cochrane RoB 2	**Low**	Strong design; directly evaluates PVP-I wound irrigation efficacy.

Table S1. Risk of Bias Assessment of Selected Studies on Cardiac Implantable Electronic Device Interventions to Prevent or Mitigate Infections.

Ref	Study	Study Type	Tool	Risk of Bias	Notes
**104**	Fjeld & Lingaas (2016)	Narrative review	N/A	**Moderate**	Focus on polyhexanide safety; descriptive, not systematic.
**105**	Luo et al. (2023)	Systematic review and meta-analysis	AMSTAR 2	**Moderate - High**	Methodologically sound, though suture technique studies are often heterogeneous.
**106**	Bou et al. (2015)	Prospective observational	ROBINS-I	**Moderate**	Focuses on absorbable subcuticular sutures; lacks randomization.
**107**	Cigna et al. (2008)	Case technique description	N/A	**High**	Innovative suture method; lacks outcome data or comparison.
**108**	Masini et al. (2011)	In vitro study	N/A	**Moderate**	Lab analysis of bacterial adherence on sutures; indirect evidence.
**109**	Spencker et al. (2010)	Randomized controlled trial (small)	Cochrane RoB 2	**Low - moderate**	Directly compares skin adhesive vs suture; some concerns due to size and scope.
**110**	Chien CY et al. (2019)	Clinical effectiveness study	ROBINS-I	**Low - moderate**	Non-randomized, likely unblinded; possible selection and detection bias.
**111**	Baldauf BJ et al. (2024)	Registry-based epidemiological study	ROBINS-I	**Low - moderate**	Large dataset reduces selection bias; potential reporting and information bias depending on data completeness.
**112**	Baldauf B et al. (2024)	Observational case series (conference abstract)	N/A	**Moderate**	Limited details; potential selection and reporting bias; lacks full peer-reviewed data.
