# Supplementary figures and images for: Addressing Complications in Cardiac Implantable Electronic Devices: A Guideline to Prevention of CIED Infection
Source: J Cardiovasc Dev Dis. 2025 Oct 13;12(10):406. doi: 10.3390/jcdd12100406 (PMC12564914; doi:10.3390/jcdd12100406)

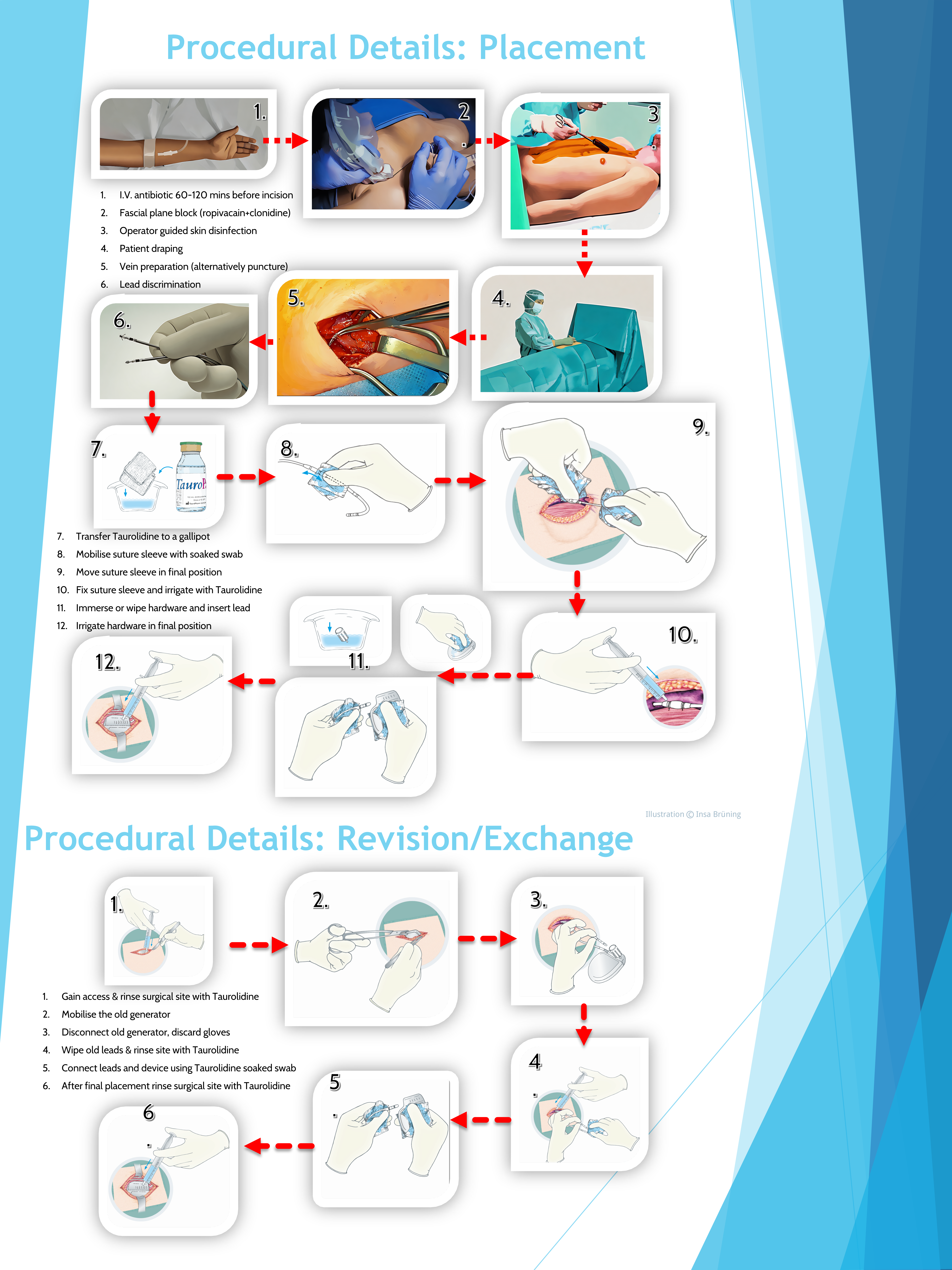

Supplement: Supplementary file 1 [file jcdd-12-00406-s001.zip › Figure_S3.tif]
